# Excitement

**Published:** 1860-03

**Authors:** 


### EXCITEMENT.

As a specimen of "the ways and means" for titillating the moral tastes of the people, we give a few of the fifty-four "Religious Notices" contained in the paper for Saturday, Feb. 11th. At the "head of the heap" is:

THE PURPLE AND FINE-LINEN GENTRY are not invited to Room No. 5, for they have received their consolation, (Luke 6 : 24;) neither are the swinish and doggish multitude, for we are commanded not to cast pearls before swine, nor to give things holy unto dogs, (Matt. 8 : 6;) nor are the Pharisees, lay and reverend, who outwardly appear to men to be righteous, (Matt. 23 : 28;) but we do invite, most cordially and respectfully, all honest and good-hearted sinners of all classes to meet us there at 10½ A.M. every SUNDAY—all who desire to understand the Scriptures of the Old and New Testaments, which can alone make a man wise unto salvation, (1 Tim. 3 : 15, 17.) The seats all free, and the teaching without money or price, (Isa. 54 : 1, 4.)

No. 2 is, "Dr. Cahill vs. Protestantism." No. 3, "Absalom, the 'Fast Young Man.'" Then another gentleman with a double D and a D. V., promises to prove that the "Roman Catholics worship Saints, Angels, and Images." Another man asks, in large letters, "Are we to continue killing people according to law?" ending with a question in small letters: "Is universal salvation possible?" Next, "The messenger of the coming Saviour holds meetings" at, etc. Another, "The friends of religious liberty are invited to hear an exiled minister of Kentucky." Another "Gent" "lectures" on "marriage." This is certain to draw the young folks who want to get married, and who would like to learn the best mode of doing it. But is this the way to "win souls"? The Reverend Mistress Blackwell Brown preaches on "Divine Impartiality." An enterprising "Baptist" preaches "at the Baptistery" on the "Glory of Baptism." There is Bap all the way through. Bapto, Baptizo, Baptos, The rear is appropriately brought up by the little end of nothing sharpened. "Andrew Jackson Davis is engaged to speak, etc. etc.," and finally "Mrs. Cora L. V. Hatch will"speak at 3½ and 7½ o'clock at, etc."

The great mass of subjects named in the fifty-four notices are of a controversial character, and assuming that all are sincere, good people, it presents a sad spectacle of brother going to war against brother; and is equal to an invitation: "Come and see how well I can fight." Hyer, Heenan, Sullivan, etc., have often given precisely the same invitation. But suppose all these men were to change their subjects to such as these, "Come and see what the Lord hath done for my soul," "Repent, for the kingdom of heaven is at hand," the burden of the whole being "Peace and good-will to men and glory to God in the highest;" if such were the tenor and spirit of every discourse, it is reasonable to suppose a much larger measure of good would be accomplished on any Sabbath day.

All this kind of thing is theological quackery, and no durable good can come of it. No man can fill his church by advertising. The very necessity of it, shows that these men have no solid reputation or ability, any more than advertising doctors have. Medical quackery brings on insidious diseases, more destructive than those they attempt to cure. Moral and religious quackery will do the same thing.

